# Interactions between heparin and SARS-CoV-2 spike glycoprotein RBD from omicron and other variants

**DOI:** 10.3389/fmolb.2022.912887

**Published:** 2022-08-15

**Authors:** Adrianne L. Gelbach, Fuming Zhang, Seok-Joon Kwon, John T. Bates, Andrew P. Farmer, Jonathan S. Dordick, Chunyu Wang, Robert J. Linhardt

**Affiliations:** ^1^ Department of Biological Sciences, Center for Biotechnology and Interdisciplinary Studies, Rensselaer Polytechnic Institute, Troy, NY, United States; ^2^ Department of Chemical and Biological Engineering, Center for Biotechnology and Interdisciplinary Studies, Rensselaer Polytechnic Institute, Troy, NY, United States; ^3^ Department of Microbiology and Immunology, University of Mississippi Medical Center, Jackson, MS, United States; ^4^ Department of Medicine, University of Mississippi Medical Center, Jackson, MS, United States; ^5^ Department of Chemistry and Chemical Biology, Center for Biotechnology and Interdisciplinary Studies, Rensselaer Polytechnic Institute, Troy, NY, United States

**Keywords:** SARS-CoV-2, spike protein RBD, heparan sulfate, heparin, surface plasmon resonance

## Abstract

Heparan sulfate (HS) acts as a co-receptor of angiotensin-converting enzyme 2 (ACE2) by interacting with severe acute respiratory syndrome-related coronavirus 2 (SARS-CoV-2) spike glycoprotein (SGP) facilitating host cell entry of SARS-CoV-2 virus. Heparin, a highly sulfated version of heparan sulfate (HS), interacts with a variety of proteins playing key roles in many physiological and pathological processes. In this study, SARS-CoV-2 SGP receptor binding domain (RBD) wild type (WT), Delta and Omicron variants were expressed in Expi293F cells and used in the kinetic and structural analysis on their interactions with heparin. Surface plasmon resonance (SPR) analysis showed the binding kinetics of SGP RBD from WT and Delta variants were very similar while Omicron variant SGP showed a much higher association rate. The SGP from Delta and Omicron showed higher affinity (*K*_*D*_) to heparin than the WT SGP. Competition SPR studies using heparin oligosaccharides indicated that binding of SGP RBDs to heparin requires chain length greater than 18. Chemically modified heparin derivatives all showed reduced interactions in competition assays suggesting that all the sulfo groups in the heparin polysaccharide were critical for binding SGP RBDs with heparin. These interactions with heparin are pH sensitive. Acidic pH (pH 6.5, 5.5, 4.5) greatly increased the binding of WT and Delta SGP RBDs to heparin, while acidic pH slightly reduced the binding of Omicron SGP RBD to heparin compared to binding at pH 7.3. In contrast, basic pH (pH 8.5) greatly reduced the binding of Omicron SGP RBDs to heparin, with much less effects on WT or Delta. The pH dependence indicates different charged residues were present at the Omicron SGP-heparin interface. Detailed kinetic and structural analysis of the interactions of SARS-CoV-2 SGP RBDs with heparin provides important information for designing anti-SARS-CoV-2 molecules.

## Introduction

Severe acute respiratory syndrome-related coronavirus 2 (SARS-CoV-2) has caused a major public health crisis, resulting in over 450 million confirmed cases of COVID-19 and over six million deaths from the disease globally, as of March 2022 (Coronavirus Resource Center, 2022). There are now several vaccines in use that target the WT SARS-CoV-2 virus spike glycoprotein (SGP), providing protection against severe illness. However, these vaccines have shown reduced effectiveness against variants of SARS-CoV-2 (Awadasseid et al., 2021; Lopez Bernal et al., 2021). Several therapeutics have been promoted to treat COVID-19, and only one drug, remdesivir (brand name Veklury), has been given FDA approval to prevent severe illness in patients who are SARS-CoV-2 positive (Awadasseid et al., 2021; FDA, 2022). There is an urgent need for effective treatments and prophylactic drugs for frontline workers and others in close contact with people who may be COVID-19 positive, as well as for those at risk of developing severe illness.

**FIGURE 1:**
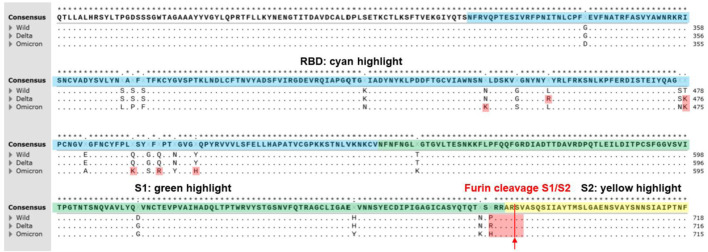
A multiple sequence alignment of SARS-CoV2 S-proteins including Alpha, Delta, and Omicron variants reproduced from CLUSTALW. Conserved sequences showed the star symbol (*) on top of consensus sequence. Yellow highlight indicates N-terminal domain (13–304, NTD). Blue highlight indicates receptor binding domain (319–540, RBD). Green highlight indicates S1 subunit (541–683). Furin cleavage sites (S1/S2 sequence) at 684 (PRRAR↓SV) were highlighted on red. Positively charged mutations in RBD (L452R and T478K at Delta variant; N440K, T478K, Q493K, Q498R, and Y505H at Omicron variant) and furin cleavage sites (P681R at Delta variant; P681H at alpha and Omicron variant) were highlighted on red. Positively charged mutations (P681H and P681R) at furin cleavage sites of SARS-CoV-2 variants contributed more efficient cleavage (**R**RRAR↓SV > **H**RRAR↓SV > **P**RRAR↓SV) resulting in increased infectivity (Lubinski et al., 2021). Positively charged mutations in RBD may contribute tighter binding to the negatively charged ridges of ACE2 around the binding site of S-protein of SARS-CoV-2 (Prabakaran et al., 2004).

The virus has continually mutated since its emergence in December 2019. Numerous mutations have been recognized by the World Health Organization (WHO) as variants of concern (VOC) and has recommended using letters of the Greek alphabet (WHO, 2022) as practical names instead of the cumbersome Pango lineage alphanumeric code (Rambaut et al., 2020). A strain must meet one or more of the following criteria to be a VOC: higher transmissibility, increased virulence, new or worsened symptoms of infection, or the measures being taken, such as vaccines, must have decreased efficacy (WHO, 2022). WHO has now declared five mutants as VOCs: Alpha, Beta, Gamma, Delta, and Omicron (WHO, 2022). The mutations in SGP for the two most common VOCs, Delta variant (prevalent until November 2021) and Omicron variant (the predominant strain after November 2021) along with the original WT amino acid sequences are shown in Figure 1 and Table 1. It is important to explore how these mutations alter the SGP interactions, which contribute to enhanced infectivity of these variants.

**TABLE 1:** SARS-CoV-2 S-protein variants. Positively charged mutations in RBD and furin cleavage sites are shown in red.

Variants	NTD (13–304)	RBD (319–540)	SD (541–683)	S2 (685–1213)
Delta India (B.1.617.2)	T19R, E156G, Δ157-158	L452R, T478K	D614G, P681R	D950N
Omicron (B.1.1.529)	67V, Δ69–70, T95I, G142D, Δ143-145, Δ211, L212I, ins214EPE	G339D, S371L, S373P, S375F, K417N, N440K, G446S, S477N, T478K, E484A, Q493K, G496S, Q498R, N501Y, Y505H	T547K, D614G, H655Y, N679K, P681H	N764K, D796Y, N856K, Q954H, N969K, L981F

SARS-CoV-2 is a *β*-coronavirus that has an RNA genome with four structural proteins: spike glycoprotein (SGP), membrane, envelope, and nucleocapsid. The SGP contains the following domains: N-terminal domain (NTD), receptor binding domain (RBD), S1/S2, S2’, fusion peptide (FP), heptad repeat 1 (HR1), central helix (CH), connector domain (CD), heptad repeat 2 (HR2), transmembrane anchor (TM), and cytoplasmic tail (CT) (Yao et al., 2020). It has been demonstrated that the SGP binds first to negatively charged glycosaminoglycans (GAGs) on the cell surface, which is then is followed by docking to angiotensin-converting enzyme 2 (ACE2) allowing the virus an entry point into the cell (Clausen et al., 2020; Kalra and Kandimalla, 2021). The RBD can exist in up or down configurations. When in the up configuration, the receptor binding motif is exposed and available to interact with the cellular receptor, ACE2, which is crucial for viral entry (Yao et al., 2020; Jackson et al., 2022). After interacting with ACE2, SARS-CoV-2 enters the cell via an endosomal pathway or by direct fusion with the cellular membrane if sufficient TMPRSS2 is available on the cell surface (Jackson et al., 2022).

GAGs are linear polysaccharides that interact with numerous proteins, playing an important role in physiology (e.g., developmental biology, wound healing, etc.) and the pathophysiology of many diseases, including cancer, cardiovascular diseases, kidney disease, skeletal diseases, eye diseases, neurological diseases, inflammation, and infectious diseases (Fromm et al., 1995; Chen et al., 1997; Linhardt and Toida, 2004; Kim et al., 2018). These are often the target of interventional therapies such as the use of heparin, chondroitin sulfate and hyaluronan [Capila and Linhardt, 2002; Linhardt, 2003). One such GAG, heparan sulfate (HS) is a linear polysaccharide attached to a core protein (proteoglycan (PG)] composed of alternating glucosamine and either iduronic or glucuronic acid residues (Linhardt, 2003). In the experiments performed, we use heparin, a closely related and highly sulfated polysaccharide, in place of heparan sulfate. The highly sulfated domains in HS known to be responsible for protein binding are also found in the more readily available anticoagulant drug, heparin (this is why HS binding proteins are often referred to as heparin-binding proteins) (Capila and Linhardt, 2002; Linhardt, 2003). The positively charged amino acids arginine (R), lysine (K), and histidine (H, at more acidic pH values) in these heparin-binding proteins interact through ion-pairing and hydrogen-bonding (Fromm et al., 1995; Hileman et al., 1998a; Hileman et al., 1998b; Capila and Linhardt, 2002) with the negatively charged groups in HS (Capila and Linhardt, 2002). HS and heparin have a high level of negative charge due to the presence of *N*- and *O*-sulfo and carboxyl groups (Linhardt and Toida, 2004; Xu and Esko, 2014). Glucosamine units can be sulfated at the 2-*N*-, 3-*O*- and 6-*O*- positions. The 2-*O*- of the glucuronic or iduronic acid units can also be sulfated. HSPGs are ubiquitous in the glycocalyx of epithelial cells in the nasal passage and act as a co-receptor in binding with SARS-CoV-2 SGP at its RBD, facilitating the conformational change in SGP necessary for binding to the ACE2 receptor (Clausen et al., 2020; Kim et al., 2022). Recently, Paiardi and coworkers reported a simulation analysis on the binding of heparin to SGP suggesting heparin inhibits SARS-CoV-2 infection by masking basic residues of both the RBD and the multifunctional S1/S2 site on SGP (Paiardi et al., 2022). Of interest, the structure of Omicron RBD has a hairpin loop in residues 369–379 which is not present in other VOCs. Omicron SGP-RBD has also been shown to have a more positive electrostatic potential than both WT and Delta (Han et al., 2022). The RBD of both WT and Delta have been shown to bind in the similar surface contacts with ACE2, however, Omicron SGP-RBD has a larger binding surface area for this receptor, indicating that perhaps the binding site for heparin could also have changes in properties among variants (Lan et al., 2022).

In our previous work, the SGP-RBD was used in surface plasmon resonance (SPR) experiments to demonstrate nanomolar binding affinity to unfractionated heparin (Tandon et al., 2021). In the current work, we examine the binding affinity of the WT, Delta, and Omicron SGP-RBD with heparin, heparin oligosaccharides of differing lengths, and chemically modified heparins using SPR to elucidate the importance of size and sulfo group positioning for HS binding. Additionally, we examined the SGP-RBD binding to heparin under at several pH values. These experiments allow a better understanding of HS co-receptor interaction with SARS-CoV-2 SGP needed to develop strategies to interfere with this initial step in viral infection.

## Materials and methods

The Omicron variant of SARS-CoV-2 SGP-RBD (B.1.1.529, Cat. # 40592-V08H121) was purchased from Sino Biological US, Inc (Wayne, PA). Based on the product information, Omicron variant of SARS-CoV-2 SGP-RBD protein construction: The DNA sequence encoding the SARS-CoV-2 Spike RBD (YP_009724390.1, with mutation G339D, S371L, S373P, S375F, K417N, N440K, G446S, S477N, T478K, E484A, Q493R, G496S, Q498R, N501Y, Y505H) (Arg319-Phe541) was expressed with a polyhistidine tag at the C-terminus. SPR measurements were performed on a Biacore T200 SPR or Biacore 3000 (Cytiva, Uppsala, Sweden). Streptavidin (SA) sensor chips and HBS-EP + buffer were purchased from Cytiva. Heparin was purchased from Celsus Laboratories (Cincinnati, OH). Heparin oligosaccharides, including hexasaccharide (degree of polymerization (dp)6), decasaccharide (dp10), tetradecasaccharide (dp14), and octadecasaccharide (dp18), and chemically modified heparins, including *N*-desulfated heparin, 2-*O*-desulfated IdoA heparin and 6-*O*-desulfated heparin, were purchased from Galen Laboratory Supplies (North Haven, CT).

### Expression and purification of SARS-CoV-2 SGP-RBD WT and delta variant in Expi293F cells

Recombinant variant RBD proteins bearing a 6x histidine tags were expressed in Expi293F cells (ThermoFisher) from constructs synthesized by Twist Biosciences that encodes amino acids 319–542 of the SARS-CoV-2 WT spike protein of the equivalent region of the variant RBDs. Expi293F cells were transfected with plasmid, and enhancers were added to the culture the following day. Cultures were grown for 6 days. Culture supernatant was harvested, centrifuged, and passed through a 0.45 um filter. Cleared supernatant was passed over a His Trap HP column (Cytiva). RBD proteins were eluted with 450 mM imidazole. Imidazole was removed via buffer exchange using a centrifugal filter device with a 10,000 Dalton cutoff (Pall Corp.).

### Preparation of heparin SPR chip

#### Biotinylation of heparin

Heparin (2 mg) and amine-PEG3-Biotin (2 mg, Pierce, Rockford, IL) were dissolved in 200 µL H_2_O and 10 mg NaCNBH_3_ was added. The reaction mixture was heated at 70°C for 24 h. An additional 10 mg NaCNBH_3_ was added and the reaction was heated at 70°C for another 24 h. The reaction solution was de-salted using a spin column (3000 molecular weight cut-off). The biotinylated heparin was freeze-dried.

#### Heparin chip preparation

In brief, a solution of biotinylated heparin (0.1 mg/ml) in HBS-EP + buffer [0.01 M 4-(2-hydroxyethyl)-1-piperazineethanesulfonic acid, 0.15 M NaCl, 3 mM ethylenediaminetetraacetic acid, 0.05% surfactant P20, pH 7.4] was injected over flow cells 2, 3, 4 (FC2, FC3, FC4) of the SA chip for 2 min at a flow rate of 10 μL/min. The successful immobilization of heparin was confirmed by the observation of a ∼200 resonance unit (RU) increase in the sensor chip. The control flow cell (FC1) was prepared by an injection of saturated biotin solution for 1 min at the same flow rate.

### Binding kinetics and affinity measurement

Each SGP-RBD sample was serially diluted into HBS-EP + buffer at concentrations of 1000, 500, 250, 125, and 63 nM. Using the Biacore T200, samples were injected into the heparin SPR chip, at a flow rate of 30 μL/min for 3 min. Following sample injection, buffer was allowed to flow over the sensor surface at the same flow rate for 3 min to facilitate dissociation. After each round, the sensor surface was regenerated by injecting 2 M NaCl for 1 min using the 30 μL/min flow rate. The response was monitored as a function of time (sensorgram) at 25°C.

### Evaluation of the inhibition activity of heparin oligosaccharides and chemically modified heparins on heparin-S-protein RBD interaction using solution competition SPR

We performed SPR competition studies between heparin bound to the chip surface and heparin analogues in solution mixed with SGP, as was carried out with many other protein-heparin interactions (Zhao et al., 2016; Zhao et al., 2020). We used both the heparin oligosaccharides with different chain length as well as the chemically desulfated heparins on the Biacore 3000 to test both the effect of chain length and sulfation pattern. To do this, SGP-RBD (125 nM) samples of each WT, Delta, and Omicron were mixed individually with 1 μM heparin oligosaccharides (dp6, dp10, dp14, dp18) or chemically desulfated heparins (2-*O*-desulfated, 6-*O*-desulated and *N*-desulfated) in HBS-EP + buffer. Samples were flowed over the heparin SPR chip at a flow rate of 30 μL/min for 3 min at 25°C. The chip was regenerated after each injection with 0.25% SDS at the same rate for 1 min. A positive control was run with each SGP-RBD mixed with heparin in HBS-EP + buffer and a negative control was run with SGP-RBD only in the same buffer.

### Effect of pH on the interaction of SGP-RBD heparin interaction

Sodium hydroxide (1 M) was used to adjust HEPES buffer + to pH 8.5 and acetic acid (1 M) was used to adjust pH to pH 5.5 and 4.5. SGP-RBDs were diluted in HBS-EP + buffer at 1 μM at five different pH values [pH 7.3 (control), 8.5, 6.5, 5.5, and 4.5] and injected onto the heparin chip at a rate of 30 μL/min on the Biacore T200 at 25°C to test the effect of pH on the SGP-RBD interaction with heparin.

## Results and discussion

### Expression and purification of SARS-CoV-2 SGP-RBD WT and delta variants in Expi293F cells

**FIGURE 2:**
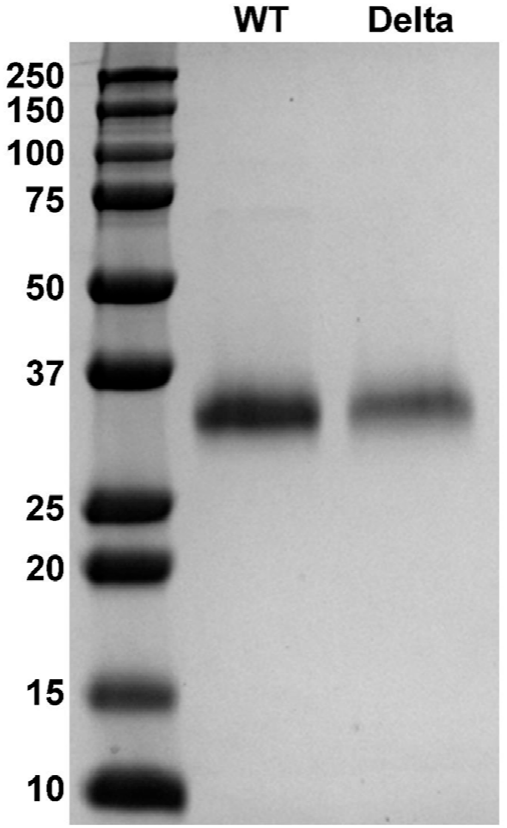
Expression and purification of SARS-CoV-2 SGP-RBD WT and Delta variant. Polyacrylamide gel electrophoresis (PAGE) images of SGP-RBD WT, and Delta variants.

Recombinant proteins of SARS-CoV-2 SGP-RBD WT and Delta variants bearing 6x-histidine tags were successfully expressed in Expi293F cells and purified with His Trap HP columns. SDS-PAGE analysis of purified protein revealed that both protein preps were >99% pure and did not exhibit any detectable impurity or degradation (Figure 2). WT RBD had a calculated molecular weight of 33.1 kDa, and Delta RBD had a calculated molecular weight of 34.0 kDa.

### Kinetic analysis on the interactions between heparin and SARS-CoV-2 SGP-RBD from Omicron and Delta variants

**FIGURE 3:**
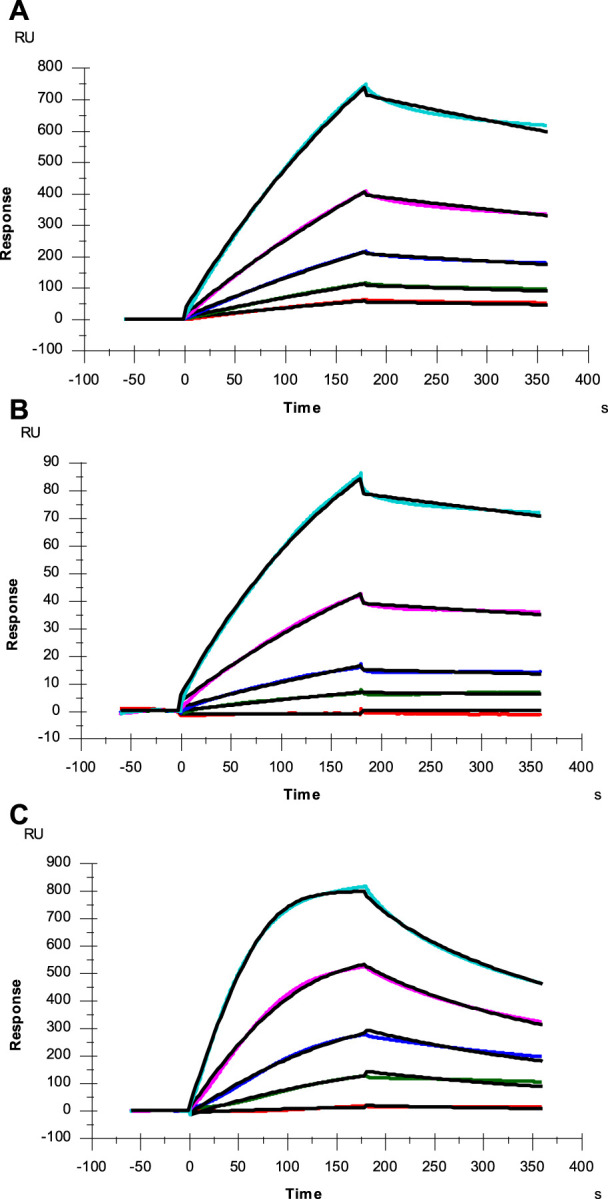
SPR sensorgrams of SGP-RBD of WT, Delta and Omicron variants interaction with heparin. Concentration of SGP-RBD mutants (from top to bottom): 1000, 500, 250, 125, and 63 nM, respectively. The black curves are the fitting curves using models from Biacore T200 Evaluation software **(A)** WT; **(B)** Delta; **(C)** Omicron.

HS interacts with SARS-CoV-2 SGP and facilitates host cell entry of SARS-CoV-2 as a co-receptor of ACE2. (Clausen et al., 2020; Kim et al., 2020; Tandon et al., 2021). As a model compound for HS, heparin has been widely used in HS-protein interaction studies. With the prevalence of new SARS-CoV-2 variants, SGP mutations have been observed, which greatly change SARS-CoV-2 infectivity, disease severity and the effectiveness of vaccines (Harvey et al., 2021). In the current study, SPR was applied to measure the binding kinetics and affinity of SARS-CoV-2 SGP-RBD (WT, Delta and Omicron) interaction with heparin using a sensor chip with immobilized heparin. Sensorgrams of SGP-RBD interactions with heparin are shown in Figure 3. Binding kinetics (i.e., association rate constant: k_a_; dissociation rate constant: k_d_) and affinity (i.e., *K*_*D*_ = k_d_/k_a_) were calculated (Table 2) by globally fitting the sensorgrams using 1:1 Langmuir binding model from T200 Evaluation software. The binding kinetics of SGP-RBD WT and Delta variants were comparable, while Omicron showed a much higher association rate than WT and Delta version, which may contribute the high infectivity of Omicron. Both Delta and Omicron SGP-RBD show higher affinity to heparin than the WT version of SGP-RBD.

**TABLE 2:** Summary of kinetic data of heparin and SARS-CoV-2 SGP-RBD (WT and mutants) interactions.^a^

Interaction	k_a_ (1/MS)	k_d_ (1/S)	Apparent K_D_ (M)
SARS-CoV-2 SGP-RBD WT	2.3 × 10^3^ (±13)	1.0 × 10^–3^ (±3.6 × 10^–6^)	4.0 × 10^–7^
SARS-CoV-2 SGP-RBD Delta	4.5 × 10^3^ (±33)	6.1 × 10^–4^ (±3.5 × 10^–6^)	1.4 × 10^–7^
SARS-CoV-2 SGP-RBD Omicron	6.0 × 10^4^ (±480)	6.3 × 10^–3^ (±3.1 × 10^–5^)	1.0 × 10^–7^

a The data with (±) in parentheses represent standard deviations (SD) from global fitting of five injections.

**FIGURE 4:**
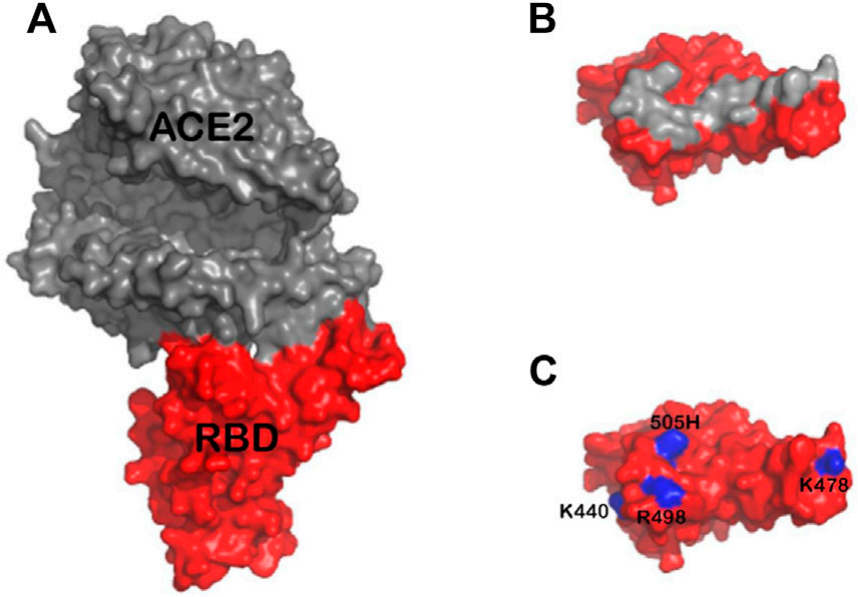
**(A)** Structure of ACE2 (gray) in complex with Omicron RBD (red) based on the previously published structure 7TN0 **(B)** Approximate footprint of the ACE2 interaction with SGP-RBD. ACE2 was removed from the image and the SGP-RBD was rotate 60°. Gray RBD residues are located within five angstroms of ACE2 in the bound structure **(C)** Residues 440K, 478K, 498R, and 505H are shown in blue. The Q493K mutation was not present in this structure.

The mutations present in the Omicron variant result in several additional positively charged amino acid residues that could play a role in the enhanced association rate (k_a)_ between Omicron RBD and heparin. However, these residues are present in or near the ACE2 binding interface (Figure 4) and not in the heparan sulfate binding site predicted by docking experiments (Clausen et al., 2020). Interestingly, Omicron RBD has a lower affinity for ACE2 than WT or Delta RBD (Wu et al., 2022). The increased k_a_ between SGP-RBD and heparan sulfate *in vivo* may compensate for the reduced affinity of the Omicron SGP-RBD interaction with ACE2 or may promote infection of cells with relatively lower levels of ACE2. *In vitro* studies of passaged viruses published before the emergence of the Omicron strain linked increased binding affinity between SGP-RBD and heparan sulfate to much higher infectivity (Shiliaev et al., 2021).

### SPR competition study with heparin oligosaccharides

**FIGURE 5:**
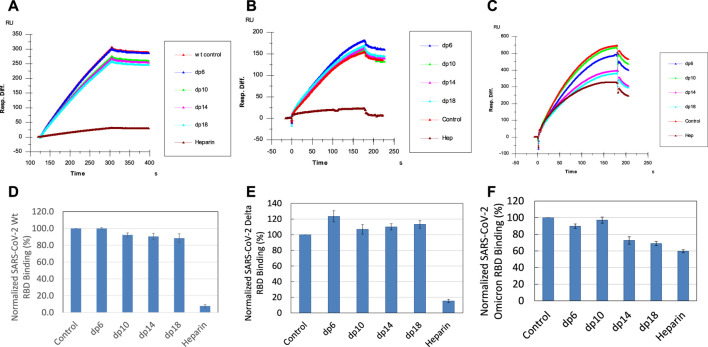
Sensorgrams and bar graphs (based on triplicate experiments with standard deviation) of normalized SGP-RBD binding to immobilized surface heparin; SGP-RBD was mixed with heparin oligosaccharides of different degrees of polymerization (dp) in solution prior to injection in direct binding competition with the surface heparin **(A)** WT; **(B)** Delta; **(C)** Omicron; **(D)** WT; **(E)** Delta; **(F)** Omicron.

SPR solution/surface competition assays were performed to test the effect of the chain length of heparin on the heparin interactions with SGP-RBDs. Different chain length heparin-derived oligosaccharides (from dp6 to dp18) at 1000 nM were applied in the competition analysis (Figure 5). For WT SGP-RBD, heparin oligosaccharides dp10, dp14 and dp18 show a weak (10–15% reduction of binding of SGP-RBD to the chip) and size-dependent inhibition on the binding. For Omicron SGP-RBD, heparin oligosaccharides dp6, dp10, dp14, and dp18 show a weak to moderate (10–30% reduction of binding of SGP-RBD to the chip) and size-dependent inhibition on the binding. Heparin in solution competed effectively against WT and Delta SGP-RBD binding to the heparin chip while less effectively inhibited Omicron SGP-RBD binding to heparin chip. To our surprise, in the case of Delta SGP-RBD, heparin oligosaccharides appeared to promote binding to chip immobilized heparin. One hypothesis for this effect may be an allosteric activation of the protein, resulting in a conformational change. This could expose an additional heparin binding site, therefore, allowing two oligosaccharides to bind the SGP-RBD, promoting binding to the immobilized heparin chip.

### SPR solution competition study on the inhibition of chemical desulfated heparin to the interaction between surface-immobilized heparin with SGP

**FIGURE 6:**
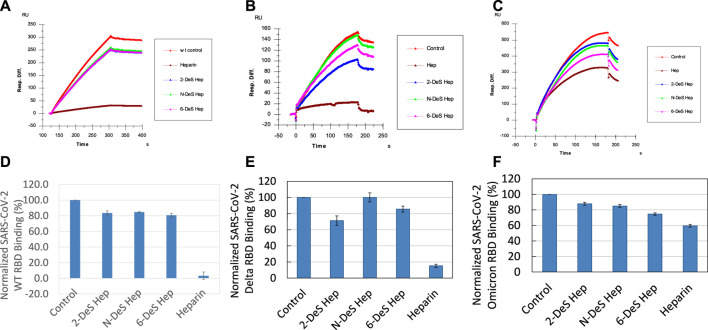
Sensorgrams and bar graphs (based on triplicate experiments with standard deviation) of normalized SGP-RBD binding to immobilized surface heparin; SGP-RBD was mixed with chemically modified heparins (2-*O*-desulfated, *N*-desulfated, and 6-*O*-desulfated) in solution prior to injection in direct binding competition with the surface heparin **(A)** WT; **(B)** Delta; **(C)** Omicron; **(D)** WT; **(E)** Delta; **(F)** Omicron.

Sulfate groups in the heparin molecule are critical for heparin-protein interactions. For example, the 3-O-sulfo group in heparin is absolutely required for its high affinity interaction with antithrombin III (Capila and Linhardt, 2002). SPR solution competition analysis was used to measure the ability of different chemically desulfated heparins to inhibit the interaction of SGP with surface-immobilized heparin. All three chemically modified heparins (N-desulfated heparin, 2-O-desulfated heparin, and 6-O-desulfated heparin) reduced the binding of all three S-protein RBDs to surface-immobilized heparin (Figure 6). The binding of WT and Delta RBD to immobilized heparin is reduced by 20–30% in the presence of desulfated heparins but were reduced by 90% in the presence of heparin (positive control). In contrast, the binding of Omicron RBD to immobilized heparin is reduced by 15–25% in the presence of desulfated heparins and reduced by only 40% in the presence of heparin. The differences between binding of heparin and desulfated heparins to S-protein RBDs are greater in WT and Delta than in Omicron suggesting that higher sulfation level in heparin may not be as important for binding Omicron SGP-RBD. These results could be due to the heparin binding loop found in Omicron SGP-RBD and positive electrostatic potential of the mutations found in Omicron making high sulfation less critical for strong interactions (Lan et al., 2022). Additionally, Omicron RBD shows less sensitive to heparin inhibition than the WT. Based on the AA sequence of the Omicron RBD (with +4 net charges than the WT), it should be more sensitive to heparin than WT in the competition assay. The unexpected results from Omicron RBD could be due to different binding kinetics of RBD towards free heparin in solution vs. immobilized heparin on chip surface. This is supported by a “classical” SPR paper reporting the large differences from values determined from chip based binding kinetics vs. affinities measured with competition SPR (Nieba et al., 1996). The binding of Omicron RBDs could be faster toward the immobilized heparin than to the solution based (competing) heparin and that once the RBD binds to the immobilized heparin the displacement becomes more difficult due to the stronger electrostatic interaction.

### Effect of pH on the SGP-RBD interaction with heparin

**FIGURE 7:**
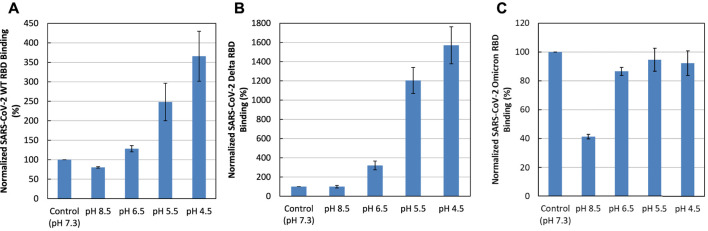
Effect of pH on the interaction of SGP-RBD interaction with heparin. Bar graphs (based on triplicate experiments with standard deviation) of normalized SGP-RBD binding to surface heparin at different pH levels **(A)** WT; **(B)** Delta; **(C)** Omicron.

Binding buffers at different pHs (pH 7.3, as a control, and pH 8.5, 6.5, 5.5, and 4.5) were used for SPR analysis to examine the effect of pH on SGP-RBD -heparin interactions. Acidic pH greatly increased the binding of WT and Delta SGP-RBDs to heparin. In contrast, lower pH slightly reduced the binding of Omicron SGP-RBD to heparin (Figure 7). Interestingly, pH 8.5 greatly reduced Omicron SGP-RBD binding to heparin but has little effects on WT or Delta. The effect of higher pH in the Omicron variant likely stems from an interfacial residue with side chain pKa near 8.5. Overall, the pH dependence indicates Omicron SGP-RBD employs different residues at its interface with heparin.

## Conclusions

In conclusion, SARS-CoV-2 SGP-RBD WT, Delta and Omicron mutants were expressed in Expi293F cells and purified for the kinetic and structural analysis on their interactions with heparin. SPR analysis revealed that the binding kinetics of SGP-RBD WT and Delta variant were comparable while Omicron showed a much higher association rate than WT and Delta variants. Both Delta and Omicron SGP-RBD showed higher affinity (*K*_*D*_) to heparin than the WT version. Competition SPR studies using heparin oligosaccharides indicated that efficient binding of SGP-RBDs requires chain length longer than 18. The testing of chemically modified heparin derivatives in the competition assays demonstrated that all the sulfation sites are important for interaction between the SGP-RBDs and heparin. Interactions are pH sensitive: acidic pH greatly increased the binding of WT and Delta SGP-RBDs to heparin while the lower pH slightly reduced the binding of Omicron SGP. Basic pH (pH 8.5) greatly reduced the binding of Omicron SGP-RBDs to heparin, with little effect in WT or Delta variant. These remarkable differences in pH dependence indicate that Omicron SGP has a different heparin interface compared with the WT or the Delta variant. The SGP-RBDs of the three variants tested showed differences in binding to heparin and its derivatives, suggesting that mutations in these variants have a profound impact on the early steps of vial attachment, possibly explaining differences in the localization and virulence of SARS-CoV-2 infection (Harvey et al., 2021). We believe the detailed kinetic and structural analysis on the interactions of SARS-CoV-2 SGP-RBDs with heparin could provide foundational information for designing anti-SARS-CoV-2 molecules.

## Data Availability

The datasets presented in this study can be found in online repositories. The names of the repository/repositories and accession number(s) can be found below: https://www.ncbi.nlm.nih.gov/, GI: 2043688783, 2106681814, and 2171220934.
