# Supplementary material for: Interactions between heparin and SARS-CoV-2 spike glycoprotein RBD from omicron and other variants
Source: Front Mol Biosci. 2022 Aug 15;9:912887. doi: 10.3389/fmolb.2022.912887 (PMC9420978; doi:10.3389/fmolb.2022.912887)
Supplement: Supplementary file 1 [file Image1.pdf]

Supporting information:

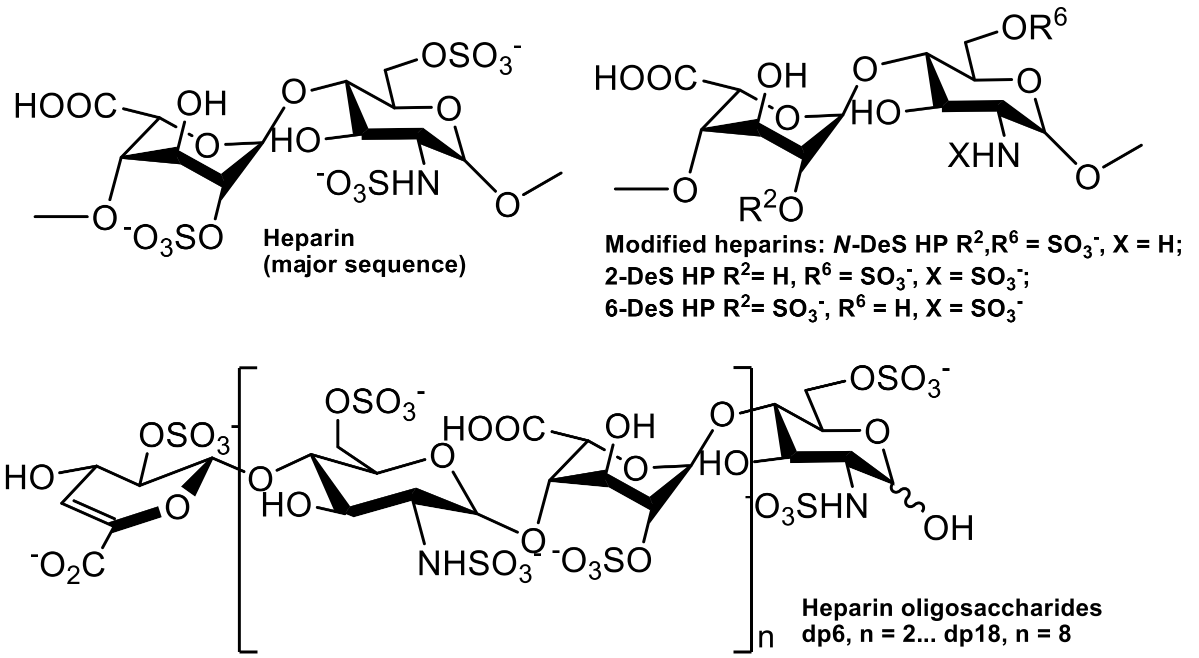

Figure S1: Chemical structures of heparin, heparin oligosaccharides and chemical modified heparins used in this study.
